# Small hyperattenuating adrenal nodules in patients with lung cancer: Differentiation of metastases from adenomas on biphasic contrast-enhanced computed tomography

**DOI:** 10.3389/fonc.2023.1091102

**Published:** 2023-02-08

**Authors:** Lixiu Cao, Libo Zhang, Wengui Xu

**Affiliations:** ^1^ Department of Molecular Imaging and Nuclear Medicine, Tianjin Medical University Cancer Institute and Hospital, National Clinical Research Center for Cancer, Tianjin Key Laboratory of Cancer Prevention and Therapy, Tianjin’s Clinical Research Center for Cancer, Tianjin, China; ^2^ Department of Emission Computed Tomography, Tangshan People’s Hospital, Tangshan, Hebei, China

**Keywords:** small hyperattenuating adrenal nodules, metastases of lung cancer, lipid-poor adenomas, biphasic contrast enhanced CT, diagnostic scoring model

## Abstract

**Objective:**

The objective of this study was to evaluate the value of biphasic contrast-enhanced computed tomography (CECT) in the differential diagnosis of metastasis and lipid-poor adenomas (LPAs) in lung cancer patients with unilateral small hyperattenuating adrenal nodule.

**Materials and methods:**

This retrospective study included 241 lung cancer patients with unilateral small hyperattenuating adrenal nodule (metastases, 123; LPAs, 118). All patients underwent plain chest or abdominal computed tomography (CT) scan and biphasic CECT scan, including arterial and venous phases. Qualitative and quantitative clinical and radiological characteristics of the two groups were compared using univariate analysis. An original diagnostic model was developed using multivariable logistic regression, and then, according to odds ratio (OR) of the risk factors of metastases, a diagnostic scoring model was developed. The areas under the receiver operating characteristic curves (AUCs) of the two diagnostic models were compared by DeLong test.

**Results:**

Compared with LAPs, metastases were older and showed more frequently irregular in shape and cystic degeneration/necrosis (all *p* < 0.05). Enhancement ratios on venous (ERV) and arterial (ERA) phase of LAPs were noticeably higher than that of metastases, whereas CT values in unenhanced phase (UP) of LPAs were noticeably lower than that of metastases (all *p* < 0.05). Compared with LAPs, the proportions of male and III/IV clinical stage and small-cell lung cancer (SCLL) were significantly higher for metastases (all *p* < 0.05). As for peak enhancement phase, LPAs showed relatively faster wash-in and earlier wash-out enhancement pattern than metastases (*p* < 0.001). Multivariate analysis revealed age ≥ 59.5 years (OR: 2.269; *p* = 0.04), male (OR: 3.511; *p* = 0.002), CT values in UP ≥ 27.5 HU (OR: 6.968; *p* < 0.001), cystic degeneration/necrosis (OR: 3.076; *p* = 0.031), ERV ≤ 1.44 (OR: 4.835; *p* < 0.001), venous phase or equally enhanced (OR: 16.907; *p* < 0.001 or OR: 14.036; *p* < 0.001), and clinical stage II or III or IV (OR: 3.550; *p* = 0.208 or OR: 17.535; *p* = 0.002 or OR: 20.241; *p* = 0.001) were risk factors for diagnosis of metastases. AUCs of the original diagnostic model and the diagnostic scoring model for metastases were 0.919 (0.883–0.955) and 0.914 (0.880–0.948), respectively. There was no statistical significance of AUC between the two diagnostic model (*p* = 0.644).

**Conclusions:**

Biphasic CECT performed well diagnostic ability in differentiating metastases from LAPs. The diagnostic scoring model is easy to popularize due to simplicity and convenience.

## 1 Introduction

Worldwide, the incidence and mortality rates of lung cancer rank first for men and second for women (1). The high mortality rate may be associated with the development of metastasis. A frequent site of metastatic spread is the adrenal glands (2). The most common malignant tumor involving the adrenal gland is metastasis, which is also the second most common adrenal tumor after adenoma. Previous studies have showed that lung cancer is the most common primary cancer of adrenal metastases (3–5); meanwhile, approximately half of adrenal tumors in patients with lung cancer were metastases (6). The qualitative diagnosis of adrenal lesions in patients with lung cancer is therefore critical to stage, direct therapy, and predict prognosis of lung cancer.

Based on endocrine function tests, clinical symptoms, and radiologic characteristics, a specific diagnosis of adrenal nodules can be achieved in many patients with lung cancer. However, when patients have nonfunctioning and indeterminate (unilateral small [long diameter, LD] ≤ 3cm) hyperattenuating [CT values in UP >10 HU]) adrenal nodules based on conventional chest or abdominal biphasic CECT, making a correct diagnosis of metastases immediately without additional diagnostic steps is challenging because the imaging features of metastases overlap with those of LPAs (7–9), especially in patients with lung cancer, and then additional confirmatory steps, such as adrenal washout CT, MRI, PET/CT, and biopsy, may be required (10–14). Although adrenal washout CT used for the characterization of adrenal nodules has relatively high sensitivity and specificity (15), it will add additional radiation and medical costs to patients, and the delay scan time is too long (10). Moreover, previous studies reported that the enhanced washout ratios of some adrenal metastases are similar to that of LPAs, leading to misdiagnosis (16). As the most sensitive examination, chemical-shift MRI still indicates indeterminate findings in approximately 10–20% of LPAs, and not all patients have high-quality MRI images (17). For PET/CT, the (18F)-fluorodeoxyglucose uptake in adenomas and metastases has a certain overlap (18). Additionally, PET/CT is not generally utilized in most institutions, and several days of waiting often occur. To achieve accurate diagnosis of indeterminate adrenal nodules, most doctors will choose invasive diagnostic procedures, such as biopsy or surgical resection, which may result in unnecessary patient anxiety and over-diagnosis and some complications (19).

Advanced image analysis technique such as radiomics has been proved to be able to differentiate adrenal tumors, especially benign and malignant lesions. However, radiomics is not used routinely in clinical practice, because it requires computational expertise and its reliability is still uncertain. Thus, there is a need for the development of simple and non-invasive rule-in or rule-out method for effectively characterizing these indeterminate adrenal nodules in lung cancer patients who await treatment for a potentially fatal disease.

Foti et al. (20) demonstrated that most adrenal metastases had a slower wash-in characteristic from biphasic CECT than adenomas. Lee et al. (21) also proved that wash-in characteristics from unenhanced to portal phase can effectively distinguish hyperattenuating adrenal tumors in lung cancer patients as to wash-out features on adrenal CT. These studies indicate that early biphasic CECT (it is equivalent to the conventional chest or abdominal biphasic CECT) without 15-min delay scan has the ability to differentiate whether adrenal lesions are metastasis or benign. In this way, not only the scanning time can be greatly reduced by omitting the 15-min delay scan but also no specific software or hardware is required to calculate the wash-in parameters based on biphasic CECT. However, previous studies only paid close attention to individual characteristic, and other quantitative and qualitative radiological characteristics were not taken into consideration for characterizing adrenal lesions. In addition, clinical stage and histologic subtypes of lung cancer were not analyzed. Moreover, data on specific “indeterminate adrenal nodules” are still very limit. Comprehensive differential diagnostic criteria may be required for “indeterminate adrenal nodules” in the characterization of lesions as metastases of lung cancer. Therefore, the purpose of the present study was to investigate whether biphasic CECT could distinguish between metastases and LAPs for indeterminate adrenal nodules in lung cancer patients.

## 2 Materials and methods

### 2.1 Patients

**Figure 1:**
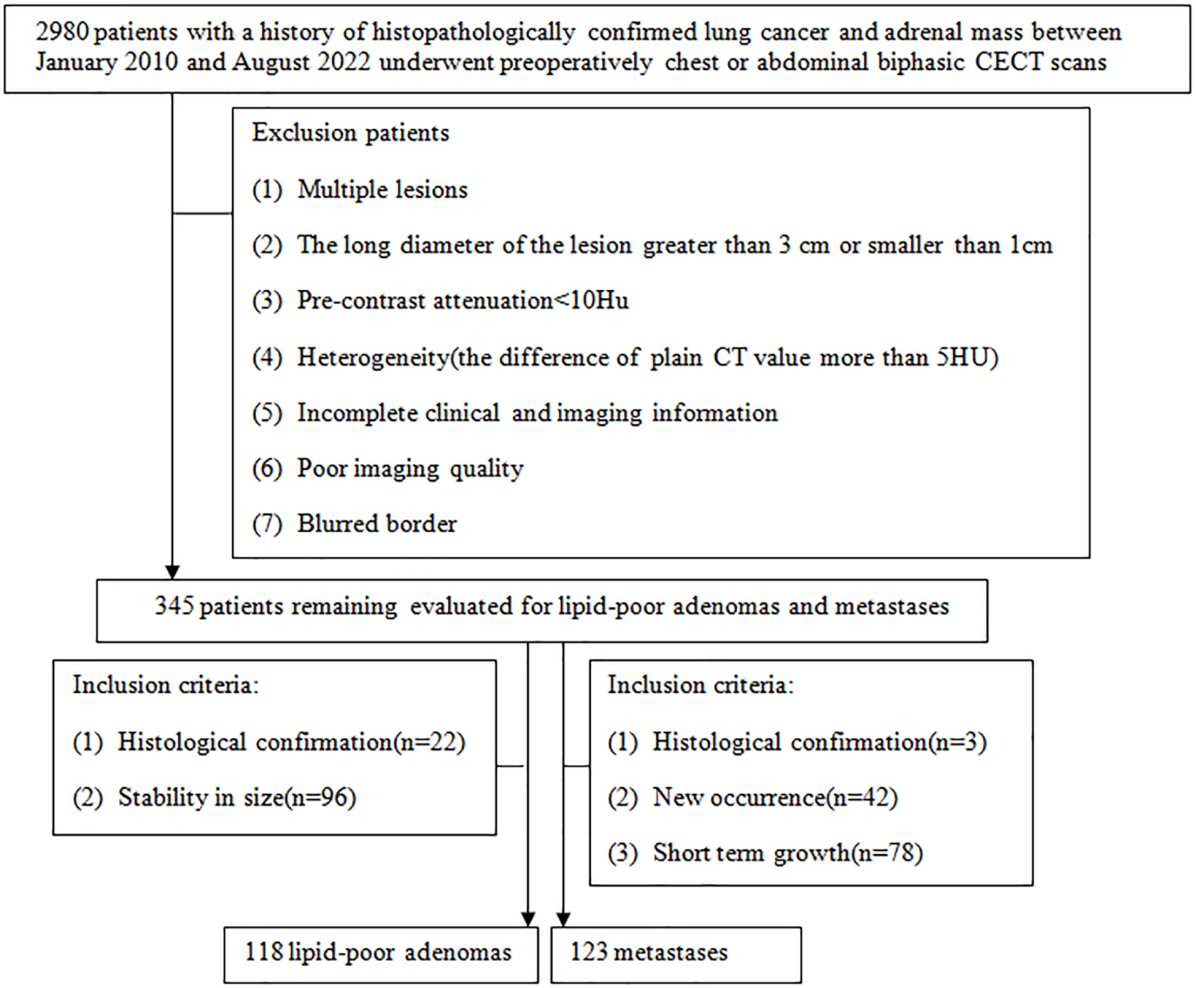
Flowchart shows the patient selection process, along with the inclusion and exclusion criteria.

This study had been approved by Tangshan People’s Hospital Institutional Ethics Committee. Patients who met the following inclusion criteria from February 2010 to August 2022 were included: (1) patients with a history of histopathologically confirmed lung cancer before or after undergoing chest or abdominal biphasic CECT; (2) indeterminate adrenal nodules: unilateral small (1 cm ≤ LD ≤ 3 cm) hyperattenuating (CT values in UP > 10 HU) adrenal nodules. There were two main reasons for the use of the cutoff of 1 cm for LD of adrenal tumor: (a) to increase confidence in the presence of a truly focal adrenal tumor and (b) to allow sufficient tumor volume for reliable quantitative measurement techniques; (3) complete clinical and imaging information. There were three eligibility criteria for diagnosing metastases: (1) histologically confirmed resection specimen or needle biopsy (*n* = 3), (2) new occurrence of a lesion in the adrenal gland on follow-up CT (*n* = 42), and (3) the total sum of the nodule of the same patient grew by 20% within 6 months (22) (*n* = 78). There were two eligibility criteria for diagnosing LAPs: (1) surgically excised and histopathological assessment (*n* = 22) and (2) stability in size after at least a 1 year interval (*n* = 96; mean follow-up time = 813 days ± 301). Finally, this study comprised 123 metastases and 118 LAPs (**Figure 1**).

### 2.2 Image protocol

Owing to retrospective analysis, this study used two CT scanners, Ingenuity core 64 (Philips Healthcare) and GE Discovery CT750 HD (GE Healthcare). All the patients undergone chest or abdominal plain and biphasic CECT scan, including arterial (approximately 30 s) and venous phase (approximately 60 s) after 80–100 ml of non-ionic contrast agent iodopamil (350 mg I/ml) was infused with a high-pressure syringe at a rate of 3.5 ml/s. The scanning parameters and image reconstruction are listed in **Supplementary Table S1**.

### 2.3 Imaging analysis

The short diameter (SD), LD, right or left, shape (regular: round or oval; irregular: unrounded), cystic degeneration/necrosis (low-density region without enhancement), CT values in UP, arterial phase (AP), and venous phase (VP) of adrenal nodules were independently measured and evaluated by two radiologists with 5 and 9 years of abdominal CT diagnosis experience on thin-sliced CT images. They were both blind to pathological results and clinical information. Disagreement was settled by consensus. LD and SD should be measured on the largest cross section of the adrenal nodules. When measuring the CT value, the region of interest (ROI) should include two-thirds of the maximum axial area of the nodules, excluding adjacent fat. In addition, we should measure three times for the attenuation values and record the average value of these values as the final result. The calculation formulas of ERA and ERV were ERA = (CT values in AP – CT values in UP)/CT values in UP and ERV = (CT values in VP –CT values in UP)/CT values in UP, respectively. We defined the phase in which maximum enhancement level was 5 HU greater than another phase as the peak enhancement phase, otherwise, equally enhanced when the difference of enhancement level was less than 5 HU between arterial and venous phase (23, 24).

### 2.4 Statistical analysis

All data were analyzed using SPSS 21, R software (version 4.2.1; http://www.rproject.org), and MedCalc 20.0.22. The χ^2^ test or Fisher’s exact test was used to compare categorical variables and the results were expressed as proportions; while continuous variables were compared using the Mann–Whitney U test or student’s t test and the results were described in mean ± standard deviation(consistent with the normal distribution) or median with interquartile range (inconsistent with the normal distribution). Every statistically significant variable was analyzed by receiver operating characteristic curve (ROC) and then the best cutoff values of quantitative variables were obtained by Youdex index for maximum specificity and sensitivity. Subsequently, the risk factors for diagnosing metastases were identified by binary logistic regression analysis. The AUC and nomograms of the original diagnostic model based on the risk factors for metastases were used to assess the diagnostic performance. According to the best cutoff values, we dichotomized the quantitative variables and then involved all risk factors into the multivariate analysis. If the risk factor was negative, the point was zero; if the risk factor was positive, the point was rounding of lnOR. At last, a diagnostic scoring model was established according to the approximate value of lnOR of risk factors for metastases. The AUC and scoring table of the diagnostic scoring model were used to show the differential diagnosis ability. DeLong test was used for the comparison between AUCs. A *p* < 0.05 was treated as significant.

## 3 Results

### 3.1 Comparison of clinical and radiological features

The mean patient age of metastases (60.9 ± 8.4 years) was significantly older than that of LAPs (55.8 ± 11.7 years) (*p* < 0.001); 75.61% of metastases (93/123) were male, whereas 39.83% of LAPs (47/118) (*p* < 0.001); 16.26% of metastases (20/123) showed irregular in shape whereas 5.93% of LAPs (7/118) did (*p* = 0.011). Cystic degeneration/necrosis was found in 25.2% of metastases (31/123) and 11.9% of LAPs (14/118), respectively (*p* = 0.008). The mean CT values in UP of metastases (37.49 ± 7.63 HU) was significantly higher than that of LAPs (27.38 ± 9.83 HU) (*p* < 0.001). The average values of ERA and ERV of metastases were 0.77 and 1.05, respectively, which were notably lower than those of LAPs (ERA: 1.60; ERV: 2.03) (all *p* < 0.001). LD, SD, Lesion location, CT values in AP, and CT values in VP were no significant differences between metastases and LAPs (all *p* > 0.05). More than half of LAPs (61.02%, 72/118) and metastases (60.98%, 75/123) were in venous phase at peak enhancement level. However, 22.03% (26/118) of LAPs were in arterial phase at peak enhancement level and only 3.25% (4/123) of metastases. While up to 35.77% (44/123) of metastases showed equally enhanced, which higher than that of LAPs. On the whole, the peak enhancement phase had significant difference between metastases and LAPs (*p* < 0.001). The highest proportion of metastases about clinical stage of lung cancer is stage IV (55.28%, 68/123), and up to 91.87% (36.59% + 55.28%) of metastases showed stages III and IV, which was significant higher than that of LAPs (61.02%, 35.60% + 25.42%); The whole clinical stage of lung cancer between LAPs and metastases was significant difference (*p* < 0.001). The proportion of small-cell lung cancer in metastases was 34.1% (42/123), which was significant higher than that of LAPs (only 8.5%, 10/118), (*p* < 0.001) (**Supplementary Table S2**).

**Figure 2:**
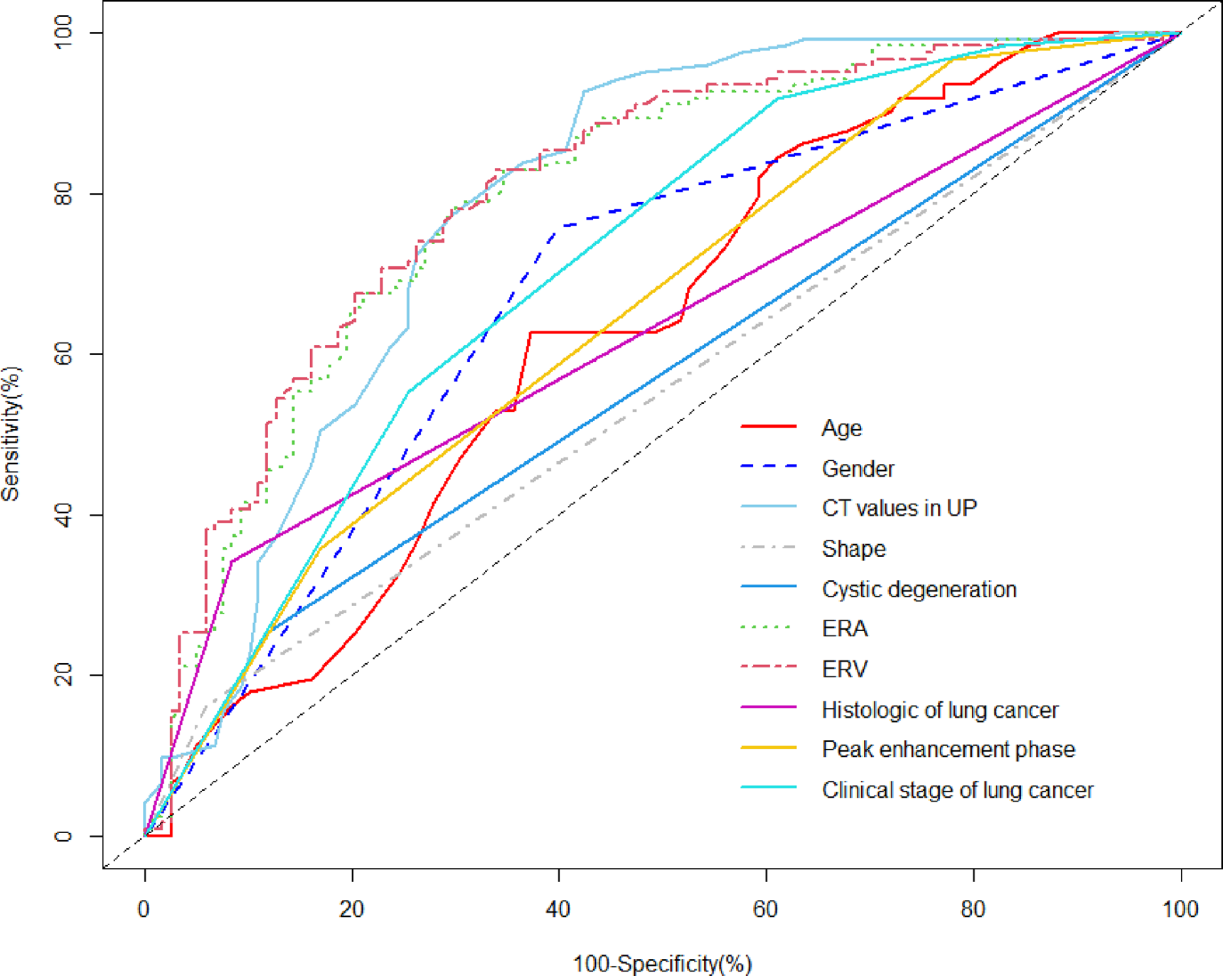
ROC analysis using significant individual variables for differentiation of metastases from LAPs. The AUC of age, CT values in UP, ERA, ERV, gender, shape, cystic degeneration, histology of lung cancer, peak enhancement phase, and clinical stage of lung cancer was 0.630, 0.789, 0.795, 0.805, 0.679, 0.552, 0.567, 0.628, 0.651, and 0.710, respectively. ERV showed a higher AUC than other individual variables.

The AUC values for quantitative and categorical variables with statistical significance were obtained by ROC analysis. Of these variables, the AUC of ERV was higher than that of age, CT values in UP, ERA, gender, shape, cystic degeneration, histology of lung cancer, peak enhancement phase, and clinical stage of lung cancer (**Table 1**; **Figure 2**). The cutoff values for age, CT values in UP, ERA, and ERV were 59.5year, 27.5 HU, 1.04, and 1.44, respectively (**Table 1**).

**Table 1:** Individual variables obtained from ROC analysis for differentiation of LAPs from metastases.

Variables	Cutoff	AUC	Sensitivity	Specificity	PPV	NPV
Age	59.5	0.630	62.6%	62.7%	63.6%	61.7%
CT values in UP	27.5	0.789	92.7%	57.6%	69.5%	88.3%
ERA	1.04	0.795	78.9%	69.5%	72.9%	75.9%
ERV	1.44	0.805	82.9%	66.1%	71.8%	78.8%
Gender	–	0.679	75.6%	60.2%	66.4%	70.3%
Shape	–	0.552	16.3%	94.1%	74.1%	51.9%
Cystic degeneration	–	0.567	25.2%	88.1%	68.9%	53.1%
Histology of lung cancer	–	0.628	34.1%	91.5%	80.8%	57.1%
Peak enhancement phase	–	0.651	96.7%	22.3%	56.4%	86.7%
Clinical stage of lung cancer	–	0.710	91.9%	39.0%	61.1%	82.1%

ROC, receiver operating characteristic curve; UP, unenhanced phase; ERA, enhancement ratio on arterial phase; ERV, enhancement ratio on venous phase; AUC, area under the curve; PPV, positive predictive value; NPV, negative predictive value.

### 3.2 Multivariate logistic regression analysis

**Figure 3:**
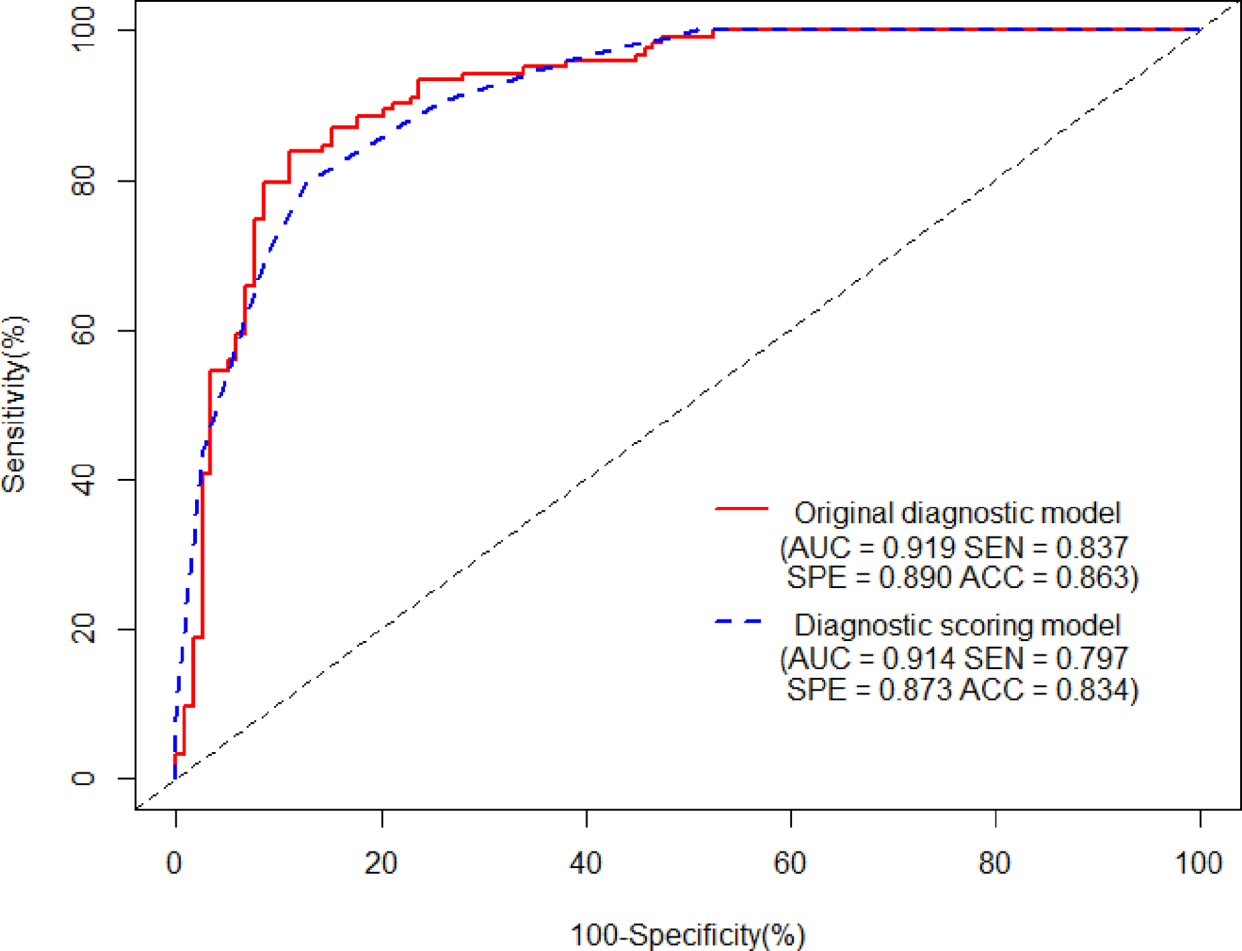
The AUCs of the original diagnostic model and the diagnostic scoring model for metastases were 0.919 (0.883–0.955) and 0.914 (0.880–0.948), respectively. There was no statistical significance of AUC between the two diagnostic model (*P* = 0.644). As for the diagnostic scoring model, a cutoff value of ≥ 9 points yielded a sensitivity of 89.43% and a specificity of 76.27% for diagnosis of metastases.

**Figure 4:**
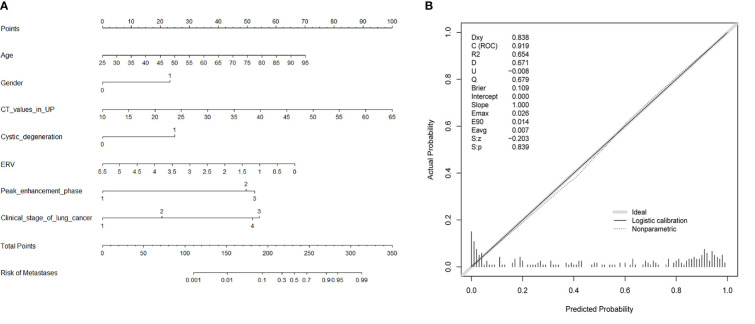
The nomogram of the original diagnostic model **(A)**. The calibration curve for nomogram **(B)**.

Ten variables—age, gender, CT values in UP, shape, cystic degeneration, ERA, ERV, peak enhancement phase, clinical stage of lung cancer, and histology of lung cancer based on univariate analysis—were further analyzed using multivariate logistic regression. Seven variables—age, gender, CT values in UP, cystic degeneration, ERV, peak enhancement phase, and clinical stage of lung cancer—were finally supposed to risk factors for differential diagnosis of metastasis and LAPs, and the original diagnostic model had high AUC of 0.919 (0.883–0.955) with ideal sensitivity (83.74%), specificity (88.98%), and accuracy (86.31%) (**Figure 3**). According to the nomogram, the probability of metastases was determined by mapping “Total Points” obtained by adding the points of each feature to the “Risk of Metastases” in the bottom of **Figure 4A**; the calibration curve for nomogram was showed in **Figure 4B**.

**Figure 5:**
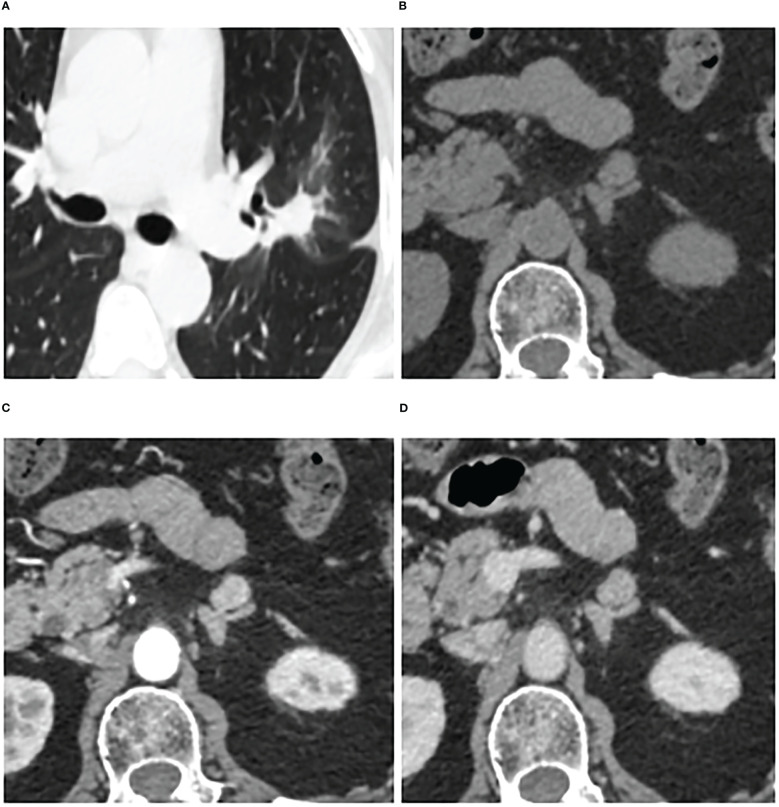
Adrenal adenoma in a 72-year-old woman. Pulmonary window of primary nonsmall-cell lung cancer (stage IV) **(A)** and unenhanced phase **(B)**, arterial phase **(C)**, and venous phase **(D)** of adrenal nodular showed a 13 mm × 11 mm tumor in left adrenal. Attenuation values on unenhanced, arterial, and venous phase were 39, 127, and 101 HU, respectively. The enhancement ratio on venous phase was 1.59. No cystic degeneration was seen within the nodular. Peak enhancement phase was arterial phase. The nodular got a score of 6 points, indicating diagnosis of adenoma.

**Figure 6:**
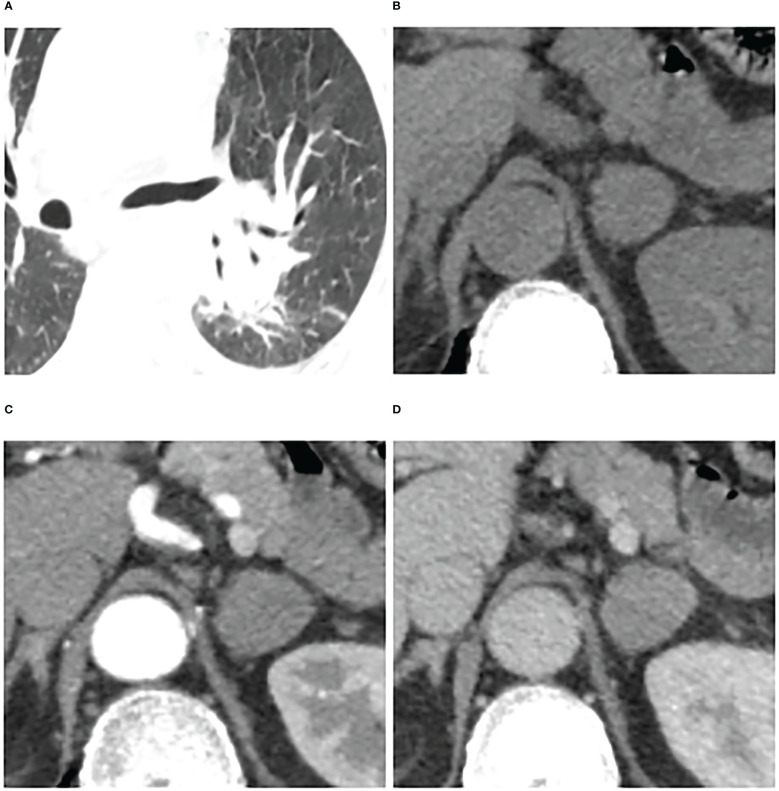
Adrenal metastases in a 66-year-old man. Pulmonary window of primary small-cell lung cancer (stage IV) **(A)** and unenhanced phase **(B)**, arterial phase **(C)**, and venous phase **(D)** of adrenal nodular showed a 25 mm × 23 mm tumor in left adrenal. Attenuation values on unenhanced, arterial, and venous phase were 36, 65, and 64 HU, respectively. The enhancement ratio on venous phase was 0.78. No cystic degeneration was seen within the nodular. Peak enhancement phase was equally enhanced. The nodular got a score of 12 points, indicating diagnosis of metastases.

**Table 2** showed the OR of all risk factors. Age ≥ 59.5years (OR: 2.269; 95% CI 1.037–4.963; *p* = 0.04), male (3.511; 95% CI 1. 1.570–7.848; *p* = 0.002), CT values in UP ≥ 27.5 HU (OR: 6.968; 95% CI 2.580–18.824; *p* < 0.001), cystic degeneration/necrosis (OR: 3.076; 95% CI 1.110–8.521; *p* = 0.031), ERV ≤ 1.44, (OR: 4.835; 95% CI 2.006–11.694; *p* < 0.001), venous phase or equally enhanced (OR: 16.907; 95% CI 4.319–66.182; *p* < 0.001 or OR: 14.036; 95% CI 3.429–57.455; *p* < 0.001), and clinical stage II or III or IV (OR: 3.550; 95% CI 0.496–25.433; *p* = 0.208 or OR: 17.535; 95% CI 2.959–103.905; *p* = 0.002 or OR: 20.241; 95% CI 3.532–116.007; *p* = 0.001) were all risk factors for the diagnosis of metastases. According to the OR of all hazard variables, a diagnostic scoring system was established and the specific score assignment was 1 point for age ≥ 59.5 years and male and cystic degeneration/necrosis and stage II, 2 points for CT values in UP ≥ 27.5HU and ERV ≤ 1.44, and 3 points for venous phase or equally enhanced and stage III or stage IV. The diagnostic scoring model also performed relatively high AUC of 0.914 (0.880–0.948) with a sensitivity of 79.67%, a specificity of 87.29%, and an accuracy of 83.40%. There was no statistical significance of AUC between the two diagnostic model (*p* = 0.644), which indicated that the diagnostic scoring model was simplified but did not affect the discriminative accuracy of metastases (**Figure 3**). **Table 3** showed the diagnostic performances with the best cutoff values. The sensitivity was 79.67% and the specificity was 87.29% when the best cutoff value ≥ 10 points. In addition, LAPs was highly hinted with the possibility as high as 93.6% when the nodule’s score was less than 8 points; metastases was highly hinted with the possibility as high as 94.7% if the score was equal to or greater than 12 points (**Table 3**). Meanwhile, the AUC of the diagnostic scoring model was significantly higher than every individual variables for differentiate metastases from LAPs (**Table 4**). Examples are given in **Figures 5**, **6**.

**Table 2:** Multivariate regression analysis for identifying metastases.

Variables	OR (95% CI)	*P* value
Age		0.040
< 59.5	1.0	
≥ 59.5	2.269 (1.031–4.963)	
Gender		0.002
Female	1.0	
Male	3.511 (1.570–7.848)	
CT values in UP		< 0.001
< 27.5	1.0	
≥ 27.5	6.968 (2.580–18.824)	
Cystic degeneration/necrosis		0.031
No	1.0	
Yes	3.076 (1.110–8.521)	
ERV		< 0.001
> 1.44	1.0	
≤ 1.44	4.835 (2.006–11.694)	
Peak enhancement phase		
Arterial phase	1.0	< 0.001
Venous phase	16.907 (4.319–66.182)	< 0.001
Equally enhanced	14.036 (3.429–57.455)	< 0.001
Clinical stage of lung cancer		
I	1.0	0.001
II	3.550 (0.496–25.433)	0.208
III	17.535 (2.959–103.905)	0.002
IV	20.241 (3.532–116.007)	0.001

OR, odds ratio; CI, confidence interval; UP, unenhanced phase; ERV, enhancement ratio on venous phase.

**Table 3:** Diagnostic performance of the diagnostic score model with different cutoffs for metastases.

Cutoff	AUC	Sensitivity(%)	Specificity(%)	PPV(%)	NPV(%)
≥2	0.5(0.5–0.5)	100(97.0–100)	0(0.0–3.1)	51.0(51.0–51.0)	
≥3	0.504(0.496–0.513)	100(97.0–100)	0.85(0.02–4.6)	51.2(50.8–51.7)	100
≥4	0.538(0.514–0.562)	100(97.0–100)	7.63(3.5–14.0)	53.0(51.7–54.3)	100
≥5	0.602(0.565–0.638)	100(97.0–100)	20.34(13.5–28.7)	56.7(54.4–58.9)	100
≥6	0.644(0.603–0.685)	100(97.0–100)	28.81(20.8–37.9)	59.4(56.6–62.2)	100
≥7	0.746(0.701–0.791)	100(97.0–100)	49.15(39.8–58.5)	67.2(63.2–71.0)	100
≥8	0.789(0.742–0.836)	95.93(90.8–98.7)	61.86(52.5–70.6)	72.4(67.5–76.8)	93.6(85.9–97.2)
≥9	0.824(0.777–0.872)	90.24(83.6–94.9)	74.58(65.7–82.1)	78.7(73.0–83.5)	88.0(80.9–92.7)
≥10	0.835(0.788–0.882)	79.67(71.5–86.4)	87.29(79.9–92.7)	86.7(80.2–91.4)	80.5(74.3–85.5)
≥11	0.803(0.755–0.851)	69.11(60.1–77.1)	91.53(85.0–95.9)	89.5(82.3–94.0)	74.0(68.5–78.8)
≥12	0.707(0.661–0.753)	43.90(35.0–53.1)	97.46(92.7–99.5)	94.7(85.3–98.2)	62.5(58.7–66.1)
≥13	0.541(0.516–0.565)	8.13(4.0–14.4)	100(96.9–100)	100	51.1(49.8–52.4)
≥14	0.5 (0.5–0.5)	0(0.0–3.0)	100(96.9–100)		49.0(49.0–49.0)

Numbers in the parentheses were 95% confidence interval; PPV, positive predictive value; NPV, negative predictive value.

**Table 4:** Comparison of performance of the diagnostic scoring model and individual variables for differentiate metastases from LAPs.

The scoring model *vs.* individual variables	AUC	Z statistic	*p*
Scoring model *vs.* age	0.914 *vs.* 0.630	7.522	< 0.001
Scoring model *vs.* CT values in UP	0.914 *vs.* 0.789	4.345	< 0.001
Scoring model *vs.* ERA	0.914 *vs.* 0.795	4.626	< 0.001
Scoring model *vs.* ERV	0.914 *vs.* 0.805	4.168	< 0.001
Scoring model *vs.* gender	0.914 *vs.* 0.679	7.488	< 0.001
Scoring model *vs.* shape	0.914 *vs.* 0.552	13.712	< 0.001
Scoring model *vs.* cystic degeneration/necrosis	0.914 *vs.* 0.567	11.882	< 0.001
Scoring model *vs.* histology of lung cancer	0.914 *vs.* 0.628	10.164	< 0.001
Scoring model *vs.* peak enhancement phase	0.914 *vs.* 0.651	8.777	< 0.001
Scoring model *vs.* clinical stage of lung cancer	0.914 *vs.* 0.710	7.074	< 0.001

AUC, area under the curve.

## Discussion

Globally, lung cancer is the main cause of cancer death (25). Almost half of lung cancer patients are found to have distant metastasis at the time of diagnosis and a frequent site of metastatic spread is the adrenal gland. Meanwhile, adrenal adenomas also have a high frequency (approximately 9%) in general population. No single imaging examination method can be regarded as the gold standard based on current studies to differentiate metastases from adenomas, especially in lung cancer cases (26). Owing to demanding time-consuming and computer expertise for the analysis of high-dimensional features that cannot be recognized by the naked eye, radiomics, as advanced image analysis techniques, has not been widely used in clinical practice. Thus, the examination and follow-up of adrenal nodules still rely mainly on the conventional imaging characteristics by visual evaluation. We evaluated the value of traditional biphasic CECT in differentiation of metastases from LAPs in this study and found that age, gender, cystic degeneration/necrosis, CT values in UP, ERV, peak enhancement phase, and clinical stage of lung cancer were risk factors for distinguishing metastases. The diagnostic scoring model established by using the above risk factors had robust ability to distinguish metastases from LAPs with AUC of 0.914 (0.880–0.948). Furthermore, compared with the original diagnostic model (or nomogram), the diagnostic scoring model (or table) had considerable differential diagnostic ability and great prospects in clinical implication because of its more simple, convenient, and accurate in the use.

We have integrated the conventional clinical and imaging variables to improve the differential diagnostic ability of biphasic CECT. Age, gender and clinical stage of lung cancer, as clinical risk variables, were all independent factors for differentiating metastases of lung cancer from LAPs in our study. Compared with LAPs, patients with metastases were more likely to be male and older. Male and a cutoff value of ≥ 59.5 years showed a higher probability for diagnosing metastases of lung cancer, which may be related to the selection of lung cancer as the primary cancer (27). Among the adrenal metastases of lung cancer, the proportions for clinical stages I and II were only 1.63% and 6.5%, respectively, whereas 36.59% and 55.28% for stages III and IV, respectively. The results were consistent with prior study (28). The later the clinical stage of lung cancer was, the higher probability adrenal nodular was to be diagnosed as metastases.

CT values in UP, the presence of cystic degeneration or necrosis, ERV, and peak enhancement phase, as CT imaging risk variables, were all independent factors for differentiating metastases of lung cancer from LAPs in our study. Compared with LAPs, metastases showed significantly higher CT values in UP and a cutoff of ≥ 28.5 HU with an OR of 1.179 in our study, consistent with the finding of Ho et al. (29), who found that there was no statistical significance of CT values in UP between benign and malignant adrenal lesions. Additionally, cystic degeneration or necrosis, reflecting the biological behavior and structural features of masses to a certain extent, was another key variable for differentiation between metastases and LAPs. The probability of cystic degeneration or necrosis in metastases was higher than LAPs, in line with previous studies (29, 30). Song et al. showed cystic degeneration or necrosis had a sensitivity of 20–55% and a specificity of 86–93% in distinguishing adrenal metastatic nodules from benign lesions (mainly adenomas) (30). The wash-in characteristics of adrenal lesions could be reflected by ERA and ERV. The ERA and ERV of metastases were notably lower than that of LAPs, and ERV was a hazard characteristic for differentiating metastasis proved by multivariate analysis (OR: 4.835; 95% CI: 2.006–11.694) in our study. The results were consistent with Lee et al. (21) who revealed that ERV of lung cancer metastases was notably lower than that of hyperattenuating benign lesions. However, Foti et al. (20) reported that the ERV of adenomas was not significantly different from metastases. The contradiction between studies may be due to differences in study cohort. First, previous studies had relatively small sample size. Second, all lesions were small (LD < 3 cm) and adenomas were all LAPs and metastases were all of lung cancer in our study. 61.02 and 22.03% of LAPs performed peak enhancement level in venous phase and arterial phase in our study, respectively, whereas 60.98% of metastases performed peak enhancement level in venous phase and only 3.25% in arterial phase and up to 35.77% equally enhanced, meaning that LAPs show relatively faster wash-in and earlier wash-out enhancement pattern than metastases, in line with previous studies (20, 31). Foti et al. (20) found that adenomas had significantly faster wash-in enhancement pattern than metastases. An et al. (31) also found up to 68.2% of LAPs had peak enhancement level in venous phase. Further studies are necessary for the difference of peak enhancement phase between LAPs and metastases of lung cancer on biphasic CECT.

Previous studies only paid close attention to individual CT radiological characteristics for differentiating metastases from adenomas. We discovered that the diagnostic accuracy was remarkably improved by combining these clinical and imaging characteristics, although these characteristics have low diagnostic specificity when used alone. The advantage of this research was that we identified independent risk factors based on large sample by multivariate analysis and established a diagnostic scoring model based on the OR by integrating above risk features. The diagnostic scoring mode performed well in differential diagnosis of metastases from LAPs with an AUC of 0.914 (0.880–0.948), which was significantly higher than that of each individual variable (*p* all < 0.05). In addition, although the performances of the original diagnostic model (nomogram) and the diagnostic scoring model (table) were similar (*p* > 0.05), we needed to obtain the points of each risk factor by visual comparison when using nomogram to predict the risk of metastases, which was prone to errors, resulting in inaccurate total points and inaccurate final prediction probability. We could directly get the points of each risk factor in the diagnostic scoring table without visual comparison. Therefore, the scoring table is more simple, convenient, and accurate in the use than nomogram, which provides an additional choice for peers.

This study had several limitations. First, our study may have some selection bias because of the retrospective nature. Second, two CT scanners and chest or abdominal CECT were used owing to the retrospective nature of our study. However, it can be considered as the strength of this research, because it conforms to the fact in work practice and provides certain potential generalizability. Third, because of lacking the delay phase, we did not estimated washout features.

In a word, biphasic CECT had well-diagnostic ability in differentiating metastases from LAPs. Age, gender, the presence of cystic degeneration, CT values in UP, ERV, peak enhancement phase, and clinical stage of lung cancer were independent risk factors for distinguishing metastases. A diagnostic scoring model integrating these risk factors with an AUC of 0.914 is easy to popularize due to simplicity and convenience.

## Data availability statement

The raw data supporting the conclusions of this article will be made available by the authors, without undue reservation.

## Ethics statement

The studies involving human participants were reviewed and approved by Tangshan People’s Hospital Institutional Ethics Committee. The patients/participants provided their written informed consent to participate in this study. Written informed consent was obtained from the individual(s) for the publication of any potentially identifiable images or data included in this article.

## Author contributions

WX, and LC together designed the study. LC collected the patient images and performed the statistical analysis and wrote the manuscript. WX and LZ critically reviewed the manuscript, LZ also provided statistical guidance. All authors contributed to the article and approved the submitted version.
