# Supplementary material for: Small hyperattenuating adrenal nodules in patients with lung cancer: Differentiation of metastases from adenomas on biphasic contrast-enhanced computed tomography
Source: Front Oncol. 2023 Feb 8;13:1091102. doi: 10.3389/fonc.2023.1091102 (PMC9972082; doi:10.3389/fonc.2023.1091102)
Supplement: Supplementary file 1 [file Table_1.docx]

Supplementary Material

# Supplementary Table 1 The scanning parameters and image reconstruction for biphasic CECT scan

|  | GE  64-MDCT  chest or abdominal biphasic CECT scan | Philips  64-MDCT  chest or abdominal biphasic CECT scan |
| --- | --- | --- |
| Tube current | Automated tube current | Automated tube current |
| Tube voltage | 120kV | 120kV |
| Reconstruction algorithm | Soft tissue standard algorithm | Soft tissue standard algorithm |
| Slice thickness | 5mm | 2mm |
| Slice increment | 5mm | 2mm |
| Reconstructed thickness | 1.25mm |  |
| Reconstructed thickness | 1.25mm |  |
| Reconstruction Matrix | 512×512 | 512×512 |
| Number of cases | 178 | 63 |

**Supplementary Table 2** Comparison of demographic characteristics and CT imaging features between LAPs and metastases

| Characteristics | LAPs | metastases | P value |
| --- | --- | --- | --- |
| Age | 55.8±11.7 | 60.9±8.4 | <0.001 |
| Gerder(male/female) | 47/71 | 93/30 | <0.001 |
| LD | 1.93±0.65 | 1.96±0.62 | 0.739 |
| SD | 1.61±0.58 | 1.58±0.54 | 0.719 |
| Lesion location(right/left) | 36/82 | 52/71 | 0.058 |
| Shape(irregular/regular) | 7/111 | 20/103 | 0.011 |
| Cystic degeneration/necrosis | 11.9%(14/118) | 25.2%(31/123) | 0.008 |
| CTU | 27.38±9.83 | 37.49±7.63 | <0.001 |
| CTA | 66.09±20.58 | 64.70±15.83 | 0.560 |
| CTV | 75.39±18.08 | 74.85±19.26 | 0.821 |
| ERA | 1.60±0.93 | 0.77±0.53 | <0.001 |
| ERV | 2.03±1.08 | 1.05±0.64 | <0.001 |
| Peak enhancement phase |  |  | <0.001 |
| Arterial phase | 22.03%(26/118) | 3.25%(4/123) |  |
| Venous phase | 61.02%(72/118) | 60.98%(75/123) |  |
| Equally enhanced | 16.95%(20/118) | 35.77%(44/123) |  |
| Clinical stage of lung cancer |  |  | <0.001 |
| I | 16.95%(20/118) | 1.63%(2/123) |  |
| II | 22.03%(26/118) | 6.50%(8/123) |  |
| III | 35.60%(42/118) | 36.59%(45/123) |  |
| IV | 25.42%(30/118) | 55.28%(68/123) |  |
| Histology of lung cancer |  |  | <0.001 |
| Small cell lung cancer | 8.5%(10/118) | 34.1%(42/123) |  |
| Non-small cell lung cancer | 91.5%(108/118) | 65.9%(81/123) |  |

LD Long diameter, SD Short diameter, CTU attenuation values on unenhanced phase, CTA attenuation values on arterial phase, CTV attenuation values on venous phase, ERA enhancement ratio on arterial phase, ERV enhancement ratio on venous phase
